# MiR-128 suppresses metastatic capacity by targeting metadherin in breast cancer cells

**DOI:** 10.1186/s40659-020-00311-5

**Published:** 2020-09-29

**Authors:** Danxia Cao, Han Zhu, Qian Zhao, Jianming Huang, Cixiang Zhou, Jianrong He, Yongjun Liang

**Affiliations:** 1 Comprehensive Breast Health Center, Shanghai Ruijin Hospital, Shanghai Jiao Tong University School of Medicine, No. 197, Rui-Jin Er Road, Shanghai, 200025 China; 2 Department of Pharmacy, Shanghai Pudong Hospital, Fudan University Pudong Medical Center, No. 2800, Gong-Wei Road, Shanghai, 201399 China; 3 Department of Pathophysiology, Key Laboratory of Cell Differentiation and Apoptosis of National Ministry of Education, Shanghai Jiao Tong University School of Medicine, No. 280, Chong-Qing South Road, Shanghai, 200025 China; 4 Department of Orthopedics, Shanghai Pudong Hospital, Fudan University Pudong Medical Center, No. 2800, Gong-Wei Road, Shanghai, 201399 China; 5 Center for Medical Research and Innovation, Shanghai Pudong Hospital, Fudan University Pudong Medical Center, No. 2800, Gong-Wei Road, Shanghai, 201399 China

**Keywords:** miR-128, Breast cancer, Metastasis, MTDH

## Abstract

**Background:**

Breast cancer, the most common cancer in women worldwide, causes the vast majority of cancer-related deaths. Undoubtedly, tumor metastasis and recurrence are responsible for more than 90 percent of these deaths. MicroRNAs are endogenous noncoding RNAs that have been integrated into almost all the physiological and pathological processes, including metastasis. In the present study, the role of miR-128 in breast cancer was investigated.

**Results:**

Compared to the corresponding adjacent normal tissue, the expression of miR-128 was significantly suppressed in human breast cancer specimens. More importantly, its expression level was reversely correlated to histological grade of the cancer. Ectopic expression of miR-128 in the aggressive breast cancer cell line MDA-MB-231 could inhibit cell motility and invasive capacity remarkably. Afterwards, Metadherin (MTDH), also known as AEG-1 (Astrocyte Elevated Gene 1) and Lyric that implicated in various aspects of cancer progression and metastasis, was further identified as a direct target gene of miR-128 and its expression level was up-regulated in clinical samples as expected. Moreover, knockdown of MTDH in MDA-MB-231 cells obviously impaired the migration and invasion capabilities, whereas re-expression of MTDH abrogated the suppressive effect caused by miR-128.

**Conclusions:**

Overall, these findings demonstrate that miR-128 could serve as a novel biomarker for breast cancer metastasis and a potent target for treatment in the future.

## Background

Breast cancer is the most common malignancy in women worldwide. Its incidence is far higher than lung and colon cancer,coming in second and third position, respectively [1]. Even more worrisome, the morbidity is still increasing. Nowadays, more than 1.7 million people are diagnosed with breast cancer globally which causes a great impact on population health [2, 3]. According to the latest statistics released by the American Cancer Society, about 268,670 new cases of invasive breast cancer are expected to be diagnosed in the United States in 2018, which means that one in eight women will get breast cancer during their lifetime [1]. In addition, 40,920 deaths per year makes breast cancer the second leading cause of cancer-related mortality among women in the USA [1]. Due to the improvements of early cancer detection and systemic therapies, breast cancer death rate has decreased in wealthy countries, such as North America and the European Union [4], while in low- and middle-income countries the situation is still urgent.

Tumor metastases are responsible for the vast majority of cancer-related deaths, and the treatments are lacking, no exception for breast cancer. Approximately 15% of patients with breast cancer suffer from distant metastatic spread, typically to bone, lung, brain and liver, and 90% of these people will eventually die of metastases [5–8]. Nevertheless, the mechanisms underlying the metastatic dissemination remain poorly understood, which causes a critical barrier for breast cancer therapy. Generally, like other solid tumors, breast cancer metastasis has a complex, multistep and multifunctional biological processes which comprises the following cascade of cellular events [9]: (1) angiogenesis, developing new blood supply to the primary tumor, (2) local invasion and migration, detaching from extracellular matrix and primary lesion, (3) intravasation, invading and entering into the blood or lymph vessels, (4) Circulation, spreading to distal organs via circulation system, (5) extravasation, invading into the endothelial cell layer, basement membrane and finally target organs, (6) colonization, proliferating to form secondary metastatic tumors. Obviously, the sequential steps mentioned above set series of natural barriers, resulting in only 0.02% of disseminated cells survived ultimately [10]. In order to overcome the extraordinarily inefficient process, numerous genetic and epigenetic events, including oncogenes activation and/or tumor suppressor genes deactivation, facilitate the breast cancer initiation and progression. For instance, aberrant expression of oncogenes, including HER2/EGFR/KRAS, SNAIL/SLUG/TWIST1, EREG/MMP1, IL6/TNFα/IL11, etc., contribute to tumour initiation, metastasis initiation, metastasis progression and metastasis virulence, respectively [11].

MicroRNAs, a class of 18–25 nucleotides noncoding RNAs, govern genes expression via binding to the 3′untranslated region (3′UTR) of mRNAs which results in translation blockade or degradation, and participate in almost all the biological events [12]. Indeed, dysregulation of microRNAs have been implicated in a broad spectrum of cellular processes underlying progression of breast cancer [13]. Recently, the metastasis-related microRNAs, whatever function as metastasis- promotor or suppressors, are collectively termed “metastamirs” [14]. More and more evidences indicate that specific spatial and temporal expression profiles of metastamirs are critical for breast cancer metastasis. As a powerful TWIST-induced microRNA, miR-10b is highly expressed in metastatic breast cancer cells and its expression level in primary breast carcinomas correlates with clinical progression. Functionally, miR-10b targets HOXD10 leading to an increase in RHOC, a pro-metastatic gene, which promotes metastasis [15]. Conversely, ectopic expression of the miR-200 family (miR-200a, miR-200b, miR-200c, miR-141 and miR-429) sufficiently reverse epithelial to mesenchymal transition (EMT) and cooperatively suppress tumor progression by inhibiting expression of the E-cadherin transcriptional repressors ZEB1/2 [16, 17]. In our previous work, a series of microRNAs with extreme low expression level in aggressive breast cancer cells were identified using microarray. One of them, miR-124, can regulate EMT and metastasis of breast cancer by targeting SLUG [18]. While another one, miR-128, was screen out with its function in breast cancer hasn’t been fully elucidated. As a neuronal-enriched microRNA, miR-128 is significantly down-regulated in glioma and inhibits cell proliferation and self-renewal [19], promotes apoptosis [20], suppresses motility [21]. Moreover, aberrant expression of miR-128 can be observed in many other malignancies, such as gastric carcinoma, lung cancer, pancreatic cancer, hepatocellular carcinoma, etc., which contributes to the tumorigenesis and metastasis [22–25]. Although few evidences show that miR-128 is involved in chemotherapeutic resistance, glucose metabolism, cell proliferation and self-renewal [26–28], its roles in breast cancer metastasis as well as the underlying mechanism have not been completely understood.

In the present study, we demonstrated that miR-128 is pathologically downregulated in breast cancer specimens and cell lines, which is reversely correlated with histological grade and cell metastatic potential, respectively. Ectopic expression of miR-128 in human breast cancer cell line MDA-MB-231 impaired cell migration and invasion capability by targeting Metadherin (MTDH). In general, these findings reveal that miR-128 plays a crucial role in breast cancer metastasis and could be a potential target for anti-metastasis therapy in the future.

## Materials and methods

### Tissue specimens and cell lines

Human breast cancer specimens and adjacent normal tissue samples were acquired from patients undergoing surgical resection in the Comprehensive Breast Health Center, Ruijin Hospital, Shanghai Jiao Tong University School of Medicine, and preserved in liquid nitrogen until use. The histological type of samples was further identified using standard hematoxylin and eosin staining. Informed consent was obtained from all patients. The study was approved by the Research Ethnics Committee of Shanghai Jiao Tong University School of Medicine.

Different cell lines were cultured under appropriate conditions as follows. Human breast cancer cell line MCF7 and immortalized human embryonic kidney cell line HEK293T were maintained in DMEM (11965092; Gibco, Carlsbad, CA, USA) with 10% FBS (10099141; Gibco, Carlsbad, CA, USA). Human breast cancer cell lines BT-474, HCC1937 and BT-549 were cultured in RPMI 1640 (11875119; Gibco, Carlsbad, CA, USA) with 10% FBS. Human breast cancer cell lines MDA-MB-468, MDA-MB-231 and MDA-MB-435 s were cultured in Leibovitz L-15 medium (11415114; Gibco, Carlsbad, CA, USA) with 10% FBS. Except for MDA-MB-468, MDA-MB-231 and MDA-MB-435 s, which were grown in humidified atmosphere of 100% air at 37°C, the others were grown in 5% CO_2_ and 95% air with the same temperature.

### RNA extraction and quantitative stem-loop reverse transcription PCR

According to the manufacturer’s instruction, total RNA was extracted using TRIzol reagent (15596018; Invitrogen, Carlsbad, CA, USA). To determine the abundance of miR-128, quantitative stem-loop reverse transcription PCR was employed. After removal of DNA contamination using RQ1 RNase-free DNase (M6101; Promega, Madison, WI, USA), cDNA was synthesized with the Prime-Script RT reagent kit (A3801; Promega, Madison, WI, USA) using the specific reaction system (Total RNA 0.625 μg in 4.25 μl, ImProm buffer 2.5 μl, dNTPs 2.5 μl, RNase inhibitor 0.625 μl, ImProm Mgcl_2_ 1.5 μl, Primer 0.5 μl, Reverse transcriptase 0.625 μl) and protocol (25 °C 5 min, 42 °C 60 min, 70 °C 15 min, 4 °C, store). Real-time PCR was performed using SYBR Green PCR Master Mix (4309155; Applied Biosystems, Foster City, CA, USA) on an Applied Biosystems 7900HT fast real-time PCR system with the following reaction system (SYBR 5.0 μl, Primer mix 0.2 μl, cDNA 1.0 μl, H_2_O 3.8 μl) and protocol (50 °C 2 min, 95 °C 5 min, (95 °C 30 s, 60 °C 40 s, 72 °C 30 s) × 40 cycles, 95 °C 15 s, 60 °C 30 s, 95 °C 15 s). Expression data were uniformly normalized to the internal control U6 and the relative expression levels were evaluated using the 2^∆∆Ct^ method. Primers for reverse transcription and real-time PCR are listed in Additional file 1: Table S1.

### Oligonucleotide transfection

The miR-128 mimics and negative control (NC) were composed of RNA duplexes with the following sense sequences. miR-128: 5ʹ-UCACAGUGAACCGGUCUCUUU-3ʹ, NC: 5ʹ-UUCUCCGAACGUGUCACGUTT-3ʹ (Genepharma, Shanghai, China). The Small interference RNAs (siRNAs) targeting MTDH were designed with the following sense sequences. siMTDH-1: 5ʹ-GGAGGAGGCUGGAAUGAAAdTdT-3ʹ, siMTDH-2: 5ʹ-CAGAUAAAUCCAAGUCAAAdTdT-3ʹ (Ribobio, Guangzhou, China). Oligoes were transfected into cells with Lipofectamine 2000 reagent (11668019; Invitrogen, Carlsbad, CA, USA) at the concentration of 100 nm.

### Cell viability assay

Cells were seeded in 96-well plates and transfected with microRNA mimics or small interference RNAs (siRNAs). Ten microliters of Cell Counting Kit-8 (96992; Sigma-Aldrich, St. Louis, MO, USA) was added to each well at 24 h, 48 h and 72 h after transfection, and then incubated for 2 h at 37 °C. The optical density at 450 nm was detected using a microplate reader.

### Wound healing assays

Cells were seeded in 24-well plates, transfected with oligoes and grown to basically 100% confluence. Then scraped acellular areas in the middle of wells were generated using sterile tips. The spread of wound closure was observed, photographed under a microscope and calculated after 24 h of serum starvation. The fraction of cell coverage across the line represents for migration rate.

### Cell migration and invasion assays

Corning Transwell Insert Chambers (3428; Corning, Tewksbury, MA, USA) and BD BioCoat Matrigel Invasion Chamber (40480; BD Biosciences, Bedford, MA, USA) were employed to evaluate the cell migration and invasion capability. Transfected cells were harvested and resuspended in serum-free medium. For migration and invasion assay, 3 × 10^4^ cells or 1 × 10^5^ cells in 200 μl medium were respectively added into the upper chamber while complete medium with FBS served as chemoattractant was added into the lower chamber. After incubation for 24 h at 37°C, cells that had migrated or invaded through the membrane were fixed with 20% methanol, stained with 0.1% crystal violet (R40052; Invitrogen, Carlsbad, CA, USA), imaged, counted and analyzed.

Cell migration and invasion assays were also carried out with a real-time cell analysis (RTCA) technology called xCELLigence^®^ system (05469759001; ACEA Biosciences, San Diego, CA, USA). The CIM-plate^®^ 16 (05665817001; ACEA Biosciences, San Diego, CA, USA) matched with RTCA DP instrument is comprised of 16 electronically integrated Boyden chambers which can make kinetic measurements of cell invasion and migration (CIM). Similar to conventional transwell assay, transfected cells were seeded into the upper chamber at 3 × 10^4^ cells per well in serum-free medium, while in the lower chamber 10% FBS contained medium was added. Prepared plates as well as instrument were placed in humidified incubator. Note that the CIM-Plate need to be precoated with 20 μl Matrigel (356234; BD Biosciences, Bedford, MA, USA) diluted by L-15 medium (1:40) for invasion assay. Data analysis was performed using RTCA software.

### Vector construction

To validate whether MTDH is a direct target of miR-128 or not, the luciferase reporter vectors hRluc-MTDH 3′UTR-WT (wild-type)/MUT (mutant) were both constructed. The wild-type 836 bp truncated 3′UTR of MTDH containing the only one conserved putative miR-128 binding site was amplified from the genomic DNA using primer pairs MTDH-UTR-F/R (Additional file 1: Table S1) and then cloned into the downstream of the Renilla luciferase (hRluc) gene in the psiCHECK™-2 Vector (C8021; Promega, Madison, WI, USA). The mutant vector containing four separate mutated bases on the predicted binding site was also constructed using the site-directed mutagenesis kit (200518; Stratagene, La Jolla, CA, USA) with primers MTDH-UTR-mutant-F/R (Additional file 1: Table S1).

For MTDH expressing vector construction, a fragment of 1814 bp was amplified with MTDH-F/R primers (Additional file 1: Table S1) from the cDNA of MCF7 cells. Using semi-nested PCR approach, 1749 bp coding region was amplified with MTDH-NF/R primers (Additional file 1: Table S1) from the 1814 bp amplicon and cloned into the pcDNA3.1 vector (V79020; Invitrogen, Carlsbad, CA, USA) by restriction enzyme XhoI and EcoRI (R0146S, R0101V; New England Biolabs, Ipswich, MA, USA).

### Luciferase assays

HEK293T cells were seeded in 24-well plates at a density of 2 × 10^5^ cells per well and allowed to grow for 24 h before transfection. 100 ng constructed hRluc-MTDH 3′UTR-WT/MUT vectors were transiently cotransfected with 60 pmol miR-128 mimic or NC into HEK293T cells using 1.44 μl Lipofectamine reagent (11668019; Invitrogen, Carlsbad, CA, USA). Cell lysates were harvested 24 h after transfection and then firefly and Renilla luciferase activities were measured by the Dual-Luciferase^®^ Reporter Assay System (E1910; Promega, Madison, WI, USA) on a Berthold AutoLumat LB9507 rack luminometer. The value of relative luciferase activity denotes the Renilla luciferase activity normalized to that of firefly for each assay.

### Western blot analysis

The standard western blotting was performed as follow. Whole cell protein lysates were electrophoresed on 10% sodium dodecyl sulfate–polyacrylamide gels and transferred onto polyvinylidene difluoride membranes (3010040001; Millipore, Burlington, MA, USA). The membranes were incubated with primary antibodies overnight at 4°C and then with the appropriate horseradish peroxidase-conjugated secondary antibody. The following antibodies were used: rabbit polyclonal antibody MTDH with working concertation 1 μg/ml (40–6400; Invitrogen, Carlsbad, CA, USA), mouse monoclonal antibody β-actin with recommended dilution 1:5000 (CP01; Millipore, Burlington, MA, USA), goat anti-rabbit secondary antibody with dilution 1:10,000 (31460; Invitrogen, Carlsbad, CA, USA), goat anti-mouse secondary antibody with dilution 1:4000 (62–6820; Invitrogen, Carlsbad, CA, USA).

### Immunohistochemistry

To visualize the expression level of MTDH in breast cancer specimens and adjacent normal tissue samples, frozen section-based immunohistochemistry was performed. The embedded specimens were sliced as 4 μm sections, dried for one hour at room temperature (RT), treated with 3% H_2_O_2_ in methanol for 10 min and then incubated with 1% bovine serum albumin (BSA) for 1 h at 37°C. The sections were incubated with primary rabbit polyclonal antibody of MTDH with the dilution 1:50 (40–6400; Invitrogen, Carlsbad, CA, USA) for 1 h at 37°C, followed by incubation with horseradish peroxidase-conjugated goat anti-rabbit secondary antibody with dilution 1:5000 (31460; Invitrogen, Carlsbad, CA, USA) at RT for 15 min. DAB and hematoxylin were used for presenting positive staining and counterstain, respectively.

### Tissue microarray

The tissue microarray with 37 human breast cancer specimens as well as paired adjacent normal tissues embedded were applied for immunohistochemistry analysis of MTDH (BR804b; Alenabio, Xi’an, China). The protocol is the same as conventional procedure (Immunohistochemistry).

### Statistical analysis

Data are presented as the mean ± SD. The differences between groups were compared using two-tailed Student’s *t* test. P < 0.05 was considered statistically significant.

## Results

### Downregulation of miR-128 in breast cancer

**Fig. 1:**
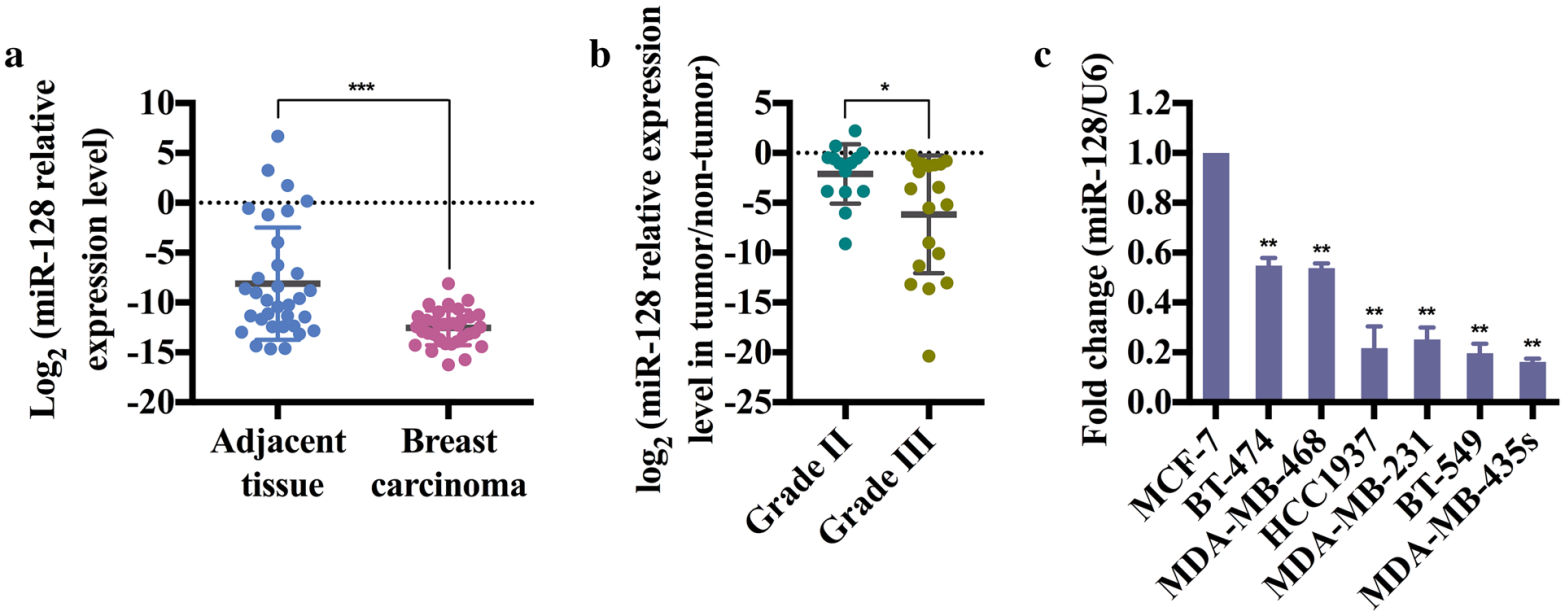
Expression of miR-128 in human breast cancer specimens and cell lines. **a** Comparison of the miR-128 abundance in 33 paired clinical cases. The miR-128 expression levels of adjacent normal tissues and cancer specimens, normalized to the internal control U6, are displayed in moderate-blue and -pink dots, respectively. **b** Comparison of the miR-128 abundance in breast cancer specimens with grade II and III. The miR-128 expression levels of breast cancer specimens with grade II (n = 14) and III (n = 19), normalized to the adjacent normal tissues, are displayed in dark-cyan and -yellow dots, respectively. **c** Difference of miR-128 in MCF7 and other six breast cancer cell lines. The expression levels of miR-128 in BT-474, MDA-MB-468, HCC1937, MDA-MB-231, BT549 and MDA-MB-435 s are normalized to MCF7, a lowest aggressive breast cancer cell line. The symbol *, ** and *** represent *P* < 0.05, *P *< 0.01 and *P *< 0.001, respectively, using a two-tailed Student’s t-test

To investigate the role of miR-128 in breast cancer progression, the expression levels between clinical breast carcinomas and paired adjacent non-neoplastic tissues from 33 cases of breast cancer patients were compared using stem-loop qRT-PCR (Additional file 1: Tables S1 and S2). Compared with adjacent normal tissues, the expression levels of miR-128 were significantly reduced in 31 of 33 cases of tumor specimens (Fig. 1a). The correlation between miR-128 expression and clinical characteristics were further analyzed (Table 1). Strikingly, the expression level of miR-128 was reversely correlated to histological grade (Fig. 1b). Considering the lower expression in tumors with grade III which means higher degree of malignancy, miR-128 could be associated with metastatic potential of breast cancer cell lines. Thus, two Luminal cell lines MCF7 and BT-474, two Basal A cell lines MDA-MB-468 and HCC1937, as well as three Basal B cell lines MDA-MB-231, BT-549 and MDA-MB-435 s were employed for evaluation of miR-128 expression levels. Undoubtedly, compared with the lowest aggressive cell line MCF7, miR-128 had lower expression levels in more malignant cell lines (Fig. 1c). Otherwise, the expression levels of miR-128 were reduced gradually from Luminal cell lines to Basal B cell lines (Fig. 1c). Overall, the reduced expression of miR-128 is a frequent event in human breast cancer, which may be involved in breast carcinoma progression, especially metastasis.

**Table 1:** Characteristics and miR-128 expression in breast cancer patients

Factors	Patients Number (%)	log_2_^(fold repression of miR−128)^ (mean ± SEM)	P value
Age(year)			0.137^a^
≤ 50	13 (39.39%)	− 2.74 ± 1.20	
> 50	20 (60.61%)	− 5.53 ± 1.24	
Tumor size(cm)			0.279^a^
≤ 3	20 (60.61%)	− 3.63 ± 1.02	
> 3	13 (39.39%)	− 5.68 ± 1.70	
Grade			0.025^a^
II	14 (42.42%)	− 2.09 ± 0.79	
III	19 (57.58%)	− 6.16 ± 1.36	
TNM stage			0.639^b^
Ι	4 (12.12%)	− 2.57 ± 1.49	
ΙΙ	22 (66.67%)	− 5.02 ± 1.23	
ΙΙΙ	7 (21.21%)	− 3.66 ± 1.72	
ER status			0.711^b^
Strongly positive	13 (39.39%)	− 5.23 ± 1.64	
Mildly positive	8 (24.24%)	− 3.24 ± 1.45	
Negative	12 (36.37)	− 4.37 ± 1.55	
PR status			0.879^b^
Strongly positive	6 (18.18%)	− 4.62 ± 2.89	
Mildly positive	11 (33.33%)	− 5.02 ± 1.47	
Negative	16 (48.49%)	− 3.96 ± 1.26	
HER2 status			0.817^b^
Strongly positive	9 (27.27%)	− 4.17 ± 2.21	
Mildly positive	20 (60.61%)	− 4.25 ± 1.08	
Negative	4 (12.12%)	− 5.97 ± 2.36	
MIB			0.805^a^
≤ 20%	15 (45.45%)	− 4.18 ± 1.39	
> 20%	18 (54.55%)	− 4.65 ± 1.24	
Node status			0.821^a^
Positive	15 (45.45%)	− 4.20 ± 1.31	
Negative	18 (54.55%)	− 4.63 ± 1.30	

*ER* Estrogen receptor, *PR* progesterone receptor

^a^T test

^b^One-way ANOVA

### miR-128 impairs migration and invasion capacity of breast cancer cell line

**Fig. 2:**
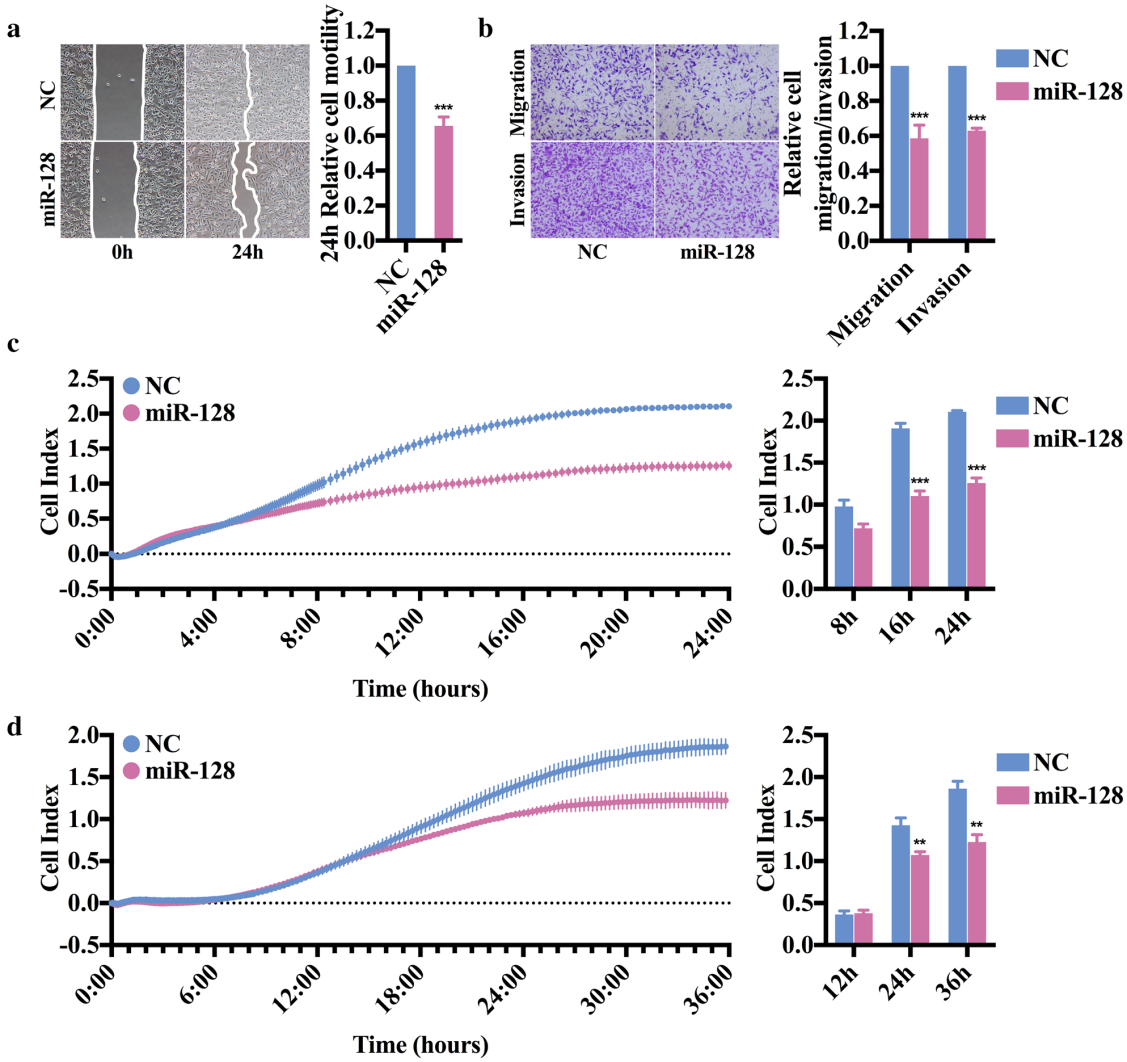
Ectopic expression of miR-128 inhibits the migration and invasion of breast cancer cell line MDA-MB-231 in vitro. **a** Wound-healing assay of MDA-MB-231 cells transfected with NC or miR-128 mimics. Representative pictures of one field at the beginning (t = 0 h) and the end of recording (t = 24 h) are shown. Bars represent the relative migrated distance of cells after scratching for 24 h. **b** Transwell migration and invasion assay of MDA-MB-231 cells transfected with NC or miR-128 mimics. Representative pictures of migrated or invaded cells with crystal violet staining are shown. Bars represent the relative migrated or invaded cells. **c** Dynamic migration assay of MDA-MB-231 cells transfected with NC or miR-128 mimics. Migration curve is formed with numerous blue (NC) or red (miR-128) dots representing cell index at different time points. Bars represent the relative migrated cells at 8 h, 16 h and 24 h. **d** Dynamic invasion assay of MDA-MB-231 cells transfected with NC or miR-128 mimics. The symbol ** and *** represent *P* < 0.01 and *P *< 0.001, respectively, using a two-tailed Student’s t-test

The lower expression levels of miR-128 in tumor specimens with higher grade and more aggressive breast cancer cell lines suggested that miR-128 downregulation may contribute to metastasis. To confirm this issue, miR-128 mimics or negative control (NC) were transiently transfected to MDA-MB-231, a highly metastatic cell line (Additional file 2: Fig. S1A). The cell viability assay showed no difference between the miR-128 and NC group, which meant that exogenous overexpression of miR-128 had no effect on proliferative capacity of MDA-MB-231 and the interference of cell proliferation on subsequent cell migration and invasion assay was excluded (Additional file 2: Fig. S1B). Afterwards, wound healing assay was carried out and revealed that ectopic expression of miR-128 dramatically inhibited cell motility compared with control group (Fig. 2a). Meanwhile, transwell migration and invasion assays demonstrated that overexpression of miR-128 in MDA-MB-231 can remarkably decrease its migration and invasion ability (Fig. 2b). The results were further confirmed by applying xCELLigence system with real-time technology which allows to observe cell migration or invasion dynamically. As expected, migration and invasion curves indicated that disparity between NC- and miR-128-transfected MDA-MB-231 cells was expanding with the extension of time (Fig. 2c, d). The cell index was extracted every 8 h for migration or 12 h for invasion and presented in Fig. 2c, d. In general, all the dates proved that miR-128 plays a critical role in breast cancer metastasis by suppressing migration and invasion.

### miR-128 directly regulates the oncogene MTDH

**Fig. 3:**
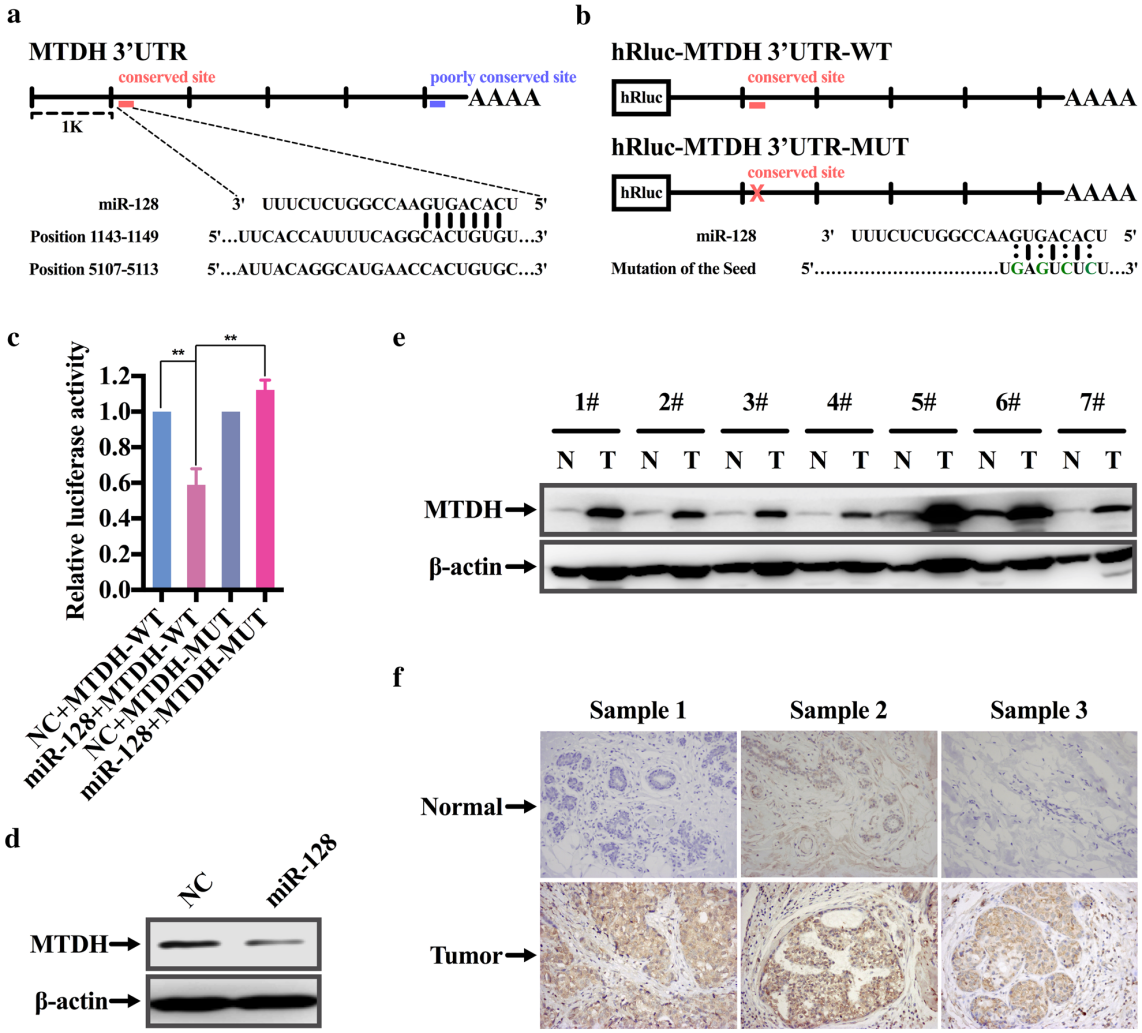
MTDH is a direct target of miR-128. **a** Schematic diagram of 3′UTR of MTDH gene and two predicted binding sites of miR-128. **b** Schematic representation of the luciferase reporter vectors including hRluc-MTDH 3′UTR-WT with wild-type binding site and hRluc-MTDH 3′UTR-WT containing four separate mutated bases in the binding site. **c** Relative activity of the luciferase gene fused with the wild-type or mutant 3′UTR of MTDH gene. **d** Western blot assay for detecting MTDH protein level of MDA-MB-231 cells transfected with NC or miR-124 mimics with β-actin served as internal control. **e** Expression of MTDH in seven paired clinical breast cancer specimens. N and T present adjacent normal tissue and paired breast cancer specimen, respectively. **f** Representative images of immunohistochemical staining for MTDH of three randomly selected clinical cases. The brown or sepia staining signal denotes MTDH-positive regions. The symbol ** represents *P *< 0.01, using a two-tailed Student’s t-test

Considering that miR-128 performed biological function indirectly, it’s important to explore and identify its target gene involved in breast cancer metastasis. Systematic in silico analyses were conducted for putative targets prediction. MTDH, an oncogene related to progression of multiple solid cancers [29, 30], was simultaneously predicted by three databases (TargetScan, PicTar and miRanda) with high rank and contained two miR-128 binding sites in its 3′UTR (Fig. 3a). In order to prove that miR-128 directly targets 3′UTR of MTDH, luciferase assay was performed. The luciferase reporter vectors hRluc-MTDH 3′UTR-WT/MUT containing the conserved binding site were constructed and transiently transfected along with miR-128 mimics or NC into HEK293 cells (Fig. 3b). Apparently, the miR-128 mimics rather than NC significantly suppressed the luciferase activity of reporter genes containing wild-type 3′UTR of MTDH, whereas the inhibition was partially rescued when the binding sites was mutated (Fig. 3c). While the luciferase assay of poorly conserved site demonstrated that miR-128 couldn’t bind to the site (data wasn’t shown). To further investigate whether miR-128 affects the expression level of MTDH, MDA-MB-231 cells were transfected with miR-128 mimics or NC. Notably, the endogenous protein level of MTDH decreased after miR-128 transfection (Fig. 3d and Additional file 3: Fig. S2A). Moreover, seven paired clinical breast cancer samples and adjacent normal tissues were randomly selected from the 33 cases, which had been used for analysis of miR-128 expression. By contrast with miR-128, MTDH was robustly increased in tumor specimens at protein level (Fig. 3e and Additional file 3: Fig. S2B). The result was confirmed by immunohistochemical staining for MTDH with representative pictures presented in Fig. 3f. In addition, a tissue microarray with 37 paraffin-embedded breast cancer specimens and paired adjacent tissue samples was employed for further validation (Additional file 1: Table S3). Similar to the previous results, the expression of MTDH was higher in tumor specimens than in normal tissues, with a positive signal detected in almost 60% of patients (Table 2 and Additional file 3: Fig. S2C). However, no correlation was found between expression of MTDH and clinical characteristics (Table 2). Taken together, miR-128 can regulate the expression of MTDH by directly targeting its 3′UTR.

**Table 2:** Characteristics and MTDH expression in breast cancer patients for tissue microarray study

Factors	MTDH			
	Negative	Positive	Positive rate (%)	P value
Type				0.019^c^
Tumor	15	22	59.46	
Peritumoral	26	11	29.73	
Age(year)				0.315^c^
< 40	5	11	68.75	
≥ 40	10	11	52.38	
Grade				1.000^c^
I	1	1	50.00	
II	11	14	56.00	
III	2	4	66.67	
Unknown	1	3		
TNM stage				0.627^c^
I/II/III	13	17	56.67	
IV	1	4	80.00	
Unknown	1	1		
Node status				
Positive	9	19	67.86	0.090^c^
Negative	5	2	28.57	
Unknown	1	1		
Histopathological subtype				1.000^c^
Ductal carcinoma	14	19	57.58	
Lobular carcinoma	1	1	50.00	
Others	0	1	100.00	
Unknown	0	1		

^c^Chi square test

### MTDH contributed to miR-128-mediated suppression of migration and invasion

**Fig. 4:**
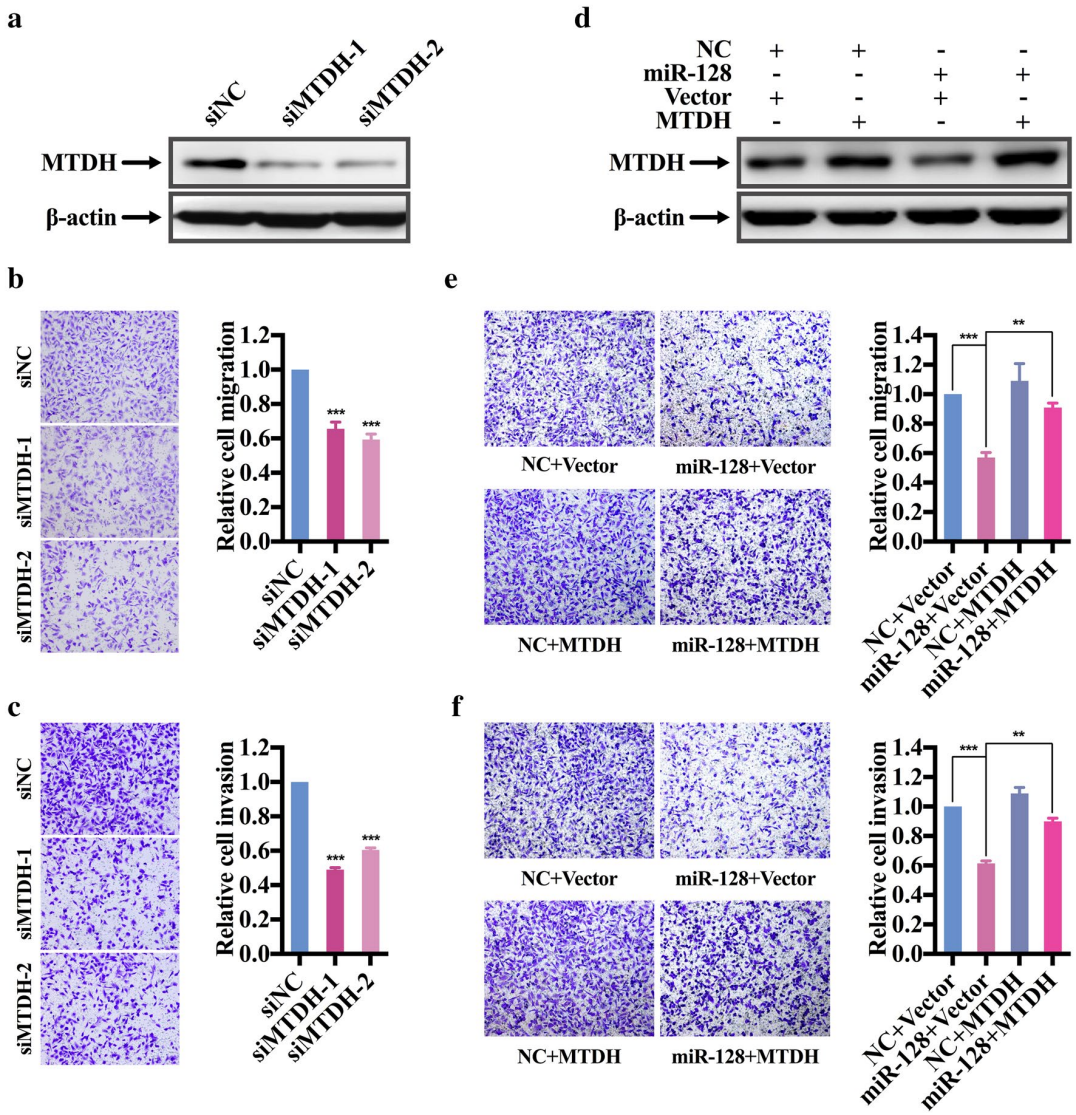
MTDH knockdown mimics miR-128-mediated phenotype and overexpression of MTDH restored miR-128-induced suppression of migration and invasion in MDA-MB-231 cells. **a** Immunoblotting analysis for expression of endogenous MTDH in MDA-MB-231 cells transfected with siNC and siMTDH-1/2. **b**, **c** Transwell migration and invasion assay of MDA-MB-231 cells transfected with siNC and siMTDH-1/2. Representative pictures of migrated or invaded cells with crystal violet staining are shown. Bars represent the relative migrated or invaded cells. **d** Western blot assay of MTDH in MDA-MB-231 cells cotransfected with NC or miR-128 mimics and pcDNA3.1-vector or pcDNA3.1-MTDH. **e**, **f** Transwell migration and invasion assay of MDA-MB-231 cells cotransfected with NC or miR-128 mimics and pcDNA3.1-vector or pcDNA3.1-MTDH. Representative pictures of migrated or invaded cells with crystal violet staining are shown. Bars represent the relative migrated or invaded cells. The symbol ** and *** represent *P* < 0.01 and *P* < 0.001, respectively, using a two-tailed Student’s t-test

To explore whether miR-128-mediated suppression of migration and invasion attributes to MTDH, two small interference RNAs directly targeting MTDH (siMTDH-1/2) were transfected into MDA-MB-231. Western blot assay showed that the two segments of siRNAs efficiently silenced the expression of MTDH (Fig. 4a and Additional file 4: Fig. S3A). Consistent with the ectopic expression of miR-128, knockdown of MTDH in MDA-MB-231 by siRNAs didn’t influence cell viability (Additional file 4: Fig. S3B), but significantly attenuated cell migration and invasion capability (Fig. 4b, c). Next, whether the restoration of MTDH can reverse the miR-128-mediated impairment of migration and invasion ability was further examined. MDA-MB-231 cells were cotransfected with miR-128 mimics or NC and pcDNA3.1-MTDH or pcDNA3.1-vector. Transfection of pcDNA3.1-MTDH in MDA-MB-231 cells efficiently rescued the low expression of MTDH caused by the high expression of miR-128 (Fig. 4d and Additional file 4: Fig. S3C). Naturally, transwell migration and invasion assay indicated that restoration of MTDH in the miR-128-transfected MDA-MB-231 cells abrogated the reduction of migration and invasion ability (Fig. 4e, f). Therefore, MTDH is a functional target involved in miR-128-mediated suppression of migration and invasion in MDA-MB-231 cells.

## Discussion

Numerous evidences have indicated that microRNAs participate in the pathogenesis of most human malignancies, including breast cancer [31]. Dysregulated microRNAs can act as tumor suppressors or oncogenes contributing to cancer initiation and progression [32, 33]. In particular, metastasis associated microRNAs whatever function as positive or negative regulators are collectively named “metastamirs” [14]. In the present study, miR-128, a metastamir, had a significant decreased expression level in human breast cancer specimens which was reversely correlated to histological grade, with lower expression levels in higher grade III. A similar phenomenon was observed that the more aggressive breast cancer cell lines had lower expression levels of miR-128. Functional studies, including wound healing assay, conventional or dynamic transwell migration and invasion assay, demonstrated that ectopic expression of miR-128 in MDA-MB-231 cells, a highly metastatic carcinoma cell line, remarkably inhibited cell migration and invasion capacity. Furthermore, MTDH, an oncogene regulating biological functions such as cell metabolism, survival, apoptosis, angiogenesis, etc. [34], was identified as a direct target gene of miR-128 and involved in miR-128-mediated suppression of migration and invasion in breast cancer cells. In conclusion, for the first time the miR-128/MTDH, a functional metastamir-oncogene pair, was proved to be a crucial regulator of breast cancer metastasis.

Metastasis accounts for predominant breast cancer mortality because of the incurable nature as well as lacking effective prevention and therapeutic approaches. In primary breast cancer, the 5-year survival rate for patients is about 93%. However, the prognosis for patients with metastasis is unfavorable, with an average 5-year survival rate dropping to 22%, despite rapid progress in adjuvant and neoadjuvant therapies [35]. Distant dissemination is a complex nonrandom multistep process known as “metastatic organotropism”, starting with detaching from primary tumor (seeds), breaking vascular wall, surviving in blood or lymph circulation, arriving target organ, adapting foreign microenvironment (congenial soil) and ending with forming metastases [36, 37]. From a macro perspective, the whole process is regulated by subtypes of breast cancer, host microenvironment, cancer cell-distant organ interactions, etc. While on the molecule level, numerous known or unknown coding or noncoding RNAs service as oncogenes or tumor suppressors and co-direct the scene of breast cancer metastasis. MicroRNAs are tiny molecules with powerful function in nearly all of biological processes including metastasis. In order to uncover the fine regulation network underlying breast cancer metastasis and provide more potential targets for future precise therapy, a number of microRNAs have been identified as metastamirs contributing to different steps. For instance, step 1) EMT as the initiation of metastasis is hindered by miR-200 family [16]; step 2) miR-145 regulates cell migration and invasion by targeting mucin-1 and c-MYC [38]; step 3) miR-26a and miR-155 inversely act on apoptosis referring to cell survival in the blood circulation [30, 39]; step 4) miR-143 disrupts cell–cell junction of vascular smooth muscle cells and enhances extravasation in vivo [40]; step 5) miR-200 s promote metastatic colonization through direct targeting of Sec23a [41]. Undoubtedly, except for these representative microRNAs listed above, there are more functional metastamirs, including the miR-128 verified in the present work, cooperating with each other and mastering breast cancer progression.

As a brain-enriched microRNA, miR-128 shows tissue- and developmental-specific expression pattern which is critical for the development of nervous system and normal physical functions maintenance [42]. Naturally, aberrant expression of miR-128 can be observed in glioma and contributes to tumorigenesis and progression. Compared with adjacent brain tissue, miR-128 is significantly reduced in glioblastoma specimens. Ectopic expression of miR-128 remarkably suppresses glioma cell proliferation in vitro and glioma xenograft growth in vivo as well as self-renewal by targeting oncogene Bmi-1 [19]. While in ATRA-induced glioblastoma cell differentiation, the expression of miR-128 is upregulated and mediate morphological changes [43]. Apoptosis of glioma cells can be also promoted by exogenous overexpression of miR-128 with its target gene RhoE downregulated [20]. Moreover, miR-128 upregulation inhibits Reelin and DCX expression leading to impairment of neuroblastoma cell motility and invasiveness [44]. Except for glioma, dysregulated miR-128 has been detected in many other types of human tumors and participates in cancer-related biological processes with different functions. In non-small cell lung cancer (NSCLC), high level of miR-128 endows mesenchymal and stemness-like properties and confers chemoresistance-associated metastasis by activating Wnt/β-catenin and TGF-β pathways [45]. Besides, both tissue and serum levels of miR-128 are decreased in prostate cancer and associated with aggressive clinicopathologic features [46]. Restored miR-128 expression improves the sensitivity of chemotherapy and inhibits the invasion capacity of prostate cancer cells [24]. Moreover, miR-128 plays important roles in squamous cell carcinomas, gastric cancer, hepatocellular carcinoma, colorectal cancer, etc. [47, 48]. Although reduced miR-128 in breast cancer has been proved to be involved in initiating cells self-renewal, chemotherapeutic resistance, cell proliferation and glucose metabolism [26–28, 49], its role in breast cancer metastasis has not been fully elucidated. In the present work, miR-128 was dramatically reduced in breast cancer specimens with its expression level reversely correlated with histological grade, a clinicopathologic feature for evaluation of metastatic tendency. Functional study demonstrated that ectopic expression of miR-128 significantly suppressed migration and invasion capacity of breast cancer cells by directly targeting oncogene MTDH. Thus, breast cancer metastasis partially attributes to the miR-128/MTDH axis.

Redundancy of pathways triggering EMT, promoting local invasion, resisting apoptosis, facilitating colonization, dominating organ-specific manner, is a hallmark of metastasis. Masses of genes collaborate with each other and activate or suppress the pathways, such as SNAI1, SULG, MMP1, CCL5, IL6, TNF, etc. MTDH, also known as astrocyte elevated gene (AEG)-1, has emerged as a primary regulator contributing to initiation and progression of various cancers, including lung cancer, gastric cancer, pancreatic cancer, colorectal cancer, prostate cancer, cervical cancer, ovarian cancer, liver cancer, etc. [34, 50–52]. Surely, MTDH also functions in several aspects of breast cancer, mainly including tumor cell proliferation [53, 54], apoptosis [30, 55], angiogenesis [56], chemoresistance [57, 58] and metastasis [59–61]. Although some studies have already demonstrated that miR-30a, miR-630, miR-320 and miR-26a can inhibit the expression of MTDH resulting in suppression of breast cancer metastasis [59–62], the “miR-128/MTDH” is a newly identified “metastamir-oncogene” pair acting on metastasis in the present work.

## Conclusions

Reduced expression of miR-128 is a frequent event in breast cancer and reversely correlated with histological grade. Ectopic expression of miR-128 can impair migration and invasion capacity of breast cancer cell by directly targeting MTDH, which makes “miR-128/MTDH” a potential contributory “metastamir-oncogene” pair for developing target therapy in the future.

## **Supplementary information**

**Additional file 1: Table S1.** Primers for miR-128 quantification, luciferase reporter plasmids and expressing vectors. **Table S2.** Clinical characteristics of breast cancer patients. **Table S3.** Clinical characteristics of patients with breast cancer for tissue microarray study.

**Additional file 2: Fig. S1.** (related to Fig. 2) Overexpression of miR-128 in breast cancer cell line MDA-MB-231 and cell viability assay after transfection. **(A)** Detection of miR-128 expression level in MDA-MB-231 cells transiently transfected with NC or miR-128 mimics. Bars represent the relative fold changes with U6 served as internal control. **(B)** Cell viability assay of MDA-MB-231 cells transfected with NC or miR-128 mimics for 24h, 48h and 72h. Bars represent the optical density at 450 nm. The symbol *** represents *P* < 0.001, using a two-tailed Student’s t-test.

**Additional file 3: Fig. S2.** (related to Fig. 3) Analysis of MTDH expression levels in breast cancer cell line, clinical specimens and tissue microarray. **(A)** The protein levels of MTDH in MDA-MB-231 cells transfected with NC or miR-128 mimics are normalized against β-actin and displayed with gray value. **(B)** The protein levels of MTDH in 7 paired clinical breast cancer specimens are normalized against β-actin and presented with gray value. **(C)** Tissue microarray for MTDH with 37 paired clinical breast cancer specimens embedded. N and T represent adjacent normal tissue and paired breast cancer specimen, respectively. The squares marked with soft blue (0) or blue (1) represent negative staining, while squares in red (2) represent positive staining. The symbol * and ** represent *P* < 0.05 and *P* < 0.01, respectively, using a two-tailed Student’s t-test.

**Additional file 4: Fig. S3.** (related to Fig. 4) Validation of MTDH knockdown and cell viability assay after MTDH silencing as well as analysis of MTDH expression levels after MTDH restoration. **(A)** The protein levels of MTDH in MDA-MB-231 cells transfected with siNC or siMTDH-1/2 are normalized against β-actin and and presented with gray value. **(B)** Cell viability assay of MDA-MB-231 cells transfected with siNC or siMTDH-1/2 for 24h, 48h and 72h. Bars represent the optical density at 450 nm. **(C)** The protein levels of MTDH in MDA-MB-231 cells cotransfected with NC or miR-128 mimics and pcDNA3.1-vector or pcDNA3.1-MTDH are normalized against β-actin and shown with gray value. The symbol *, ** and *** represent *P* < 0.05, *P* < 0.01 and *P* < 0.001, respectively, using a two-tailed Student’s t-test.

## Data Availability

The datasets used and/or analyzed during the current study are available from the corresponding author on reasonable request.

## Abbreviations

qRT-PCR: Quantitative real-time polymerase chain reaction RT-PCR
NC: Negative control
MTDH: Metadherin
3′UTR: 3′untranslated region
EMT: Epithelial to mesenchymal transition
siRNAs: Small interference RNAs
