# Supplementary material for: MiR-128 suppresses metastatic capacity by targeting metadherin in breast cancer cells
Source: Biol Res. 2020 Sep 29;53:43. doi: 10.1186/s40659-020-00311-5 (PMC7526227; doi:10.1186/s40659-020-00311-5)
Supplement: Supplementary file 1 — Additional file 1: Table S1. Primers for miR-128 quantification, luciferase reporter plasmids and expressing vectors. Table S2. Clinical characteristics of breast cancer patients. Table S3. Clinical characteristics of patients with breast cancer for tissue microarray study. [file 40659_2020_311_MOESM1_ESM.doc]

Table S1. Primers for miR-128 quantification, luciferase reporter plasmids and expressing vectors

| **Name** | **Sequence (5’-3’)** |
| --- | --- |
| miR-128-RT | GTCGTATCCAGTGCAGGGTCCGAGGTATTCGCACTGGATACGACAAAGAG |
| miR-128-F | GGACCTCACAGTGAACCG |
| miR-128-R | GTGCAGGGTCCGAGGT |
| U6-F | CGCTTCGGCAGCACATATAC |
| U6-R | CAGGGGCCATGCTAATCTT |
| MTDH-UTR-F | CCGCTCGAGTTAACCTGTAGTGCGTAGAATATG |
| MTDH-UTR-R | ATTTGCGGCCGCAAATTAGATTCCCTATCAACTTCTC |
| MTDH-UTR-mutant-F | GTTTCACCATTTTCAGGGAGTCTCTAATTCTATTGTAATAAACTGGC |
| MTDH-UTR-mutant-R | GCCAGTTTATTACAATAGAATTAGAGACTCCCTGAAAATGGTGAAAC |
| MTDH-F | TCGACTATTCCACTGCGTCTCCG |
| MTDH-NF | ATGGCTGCACGGAGCTGGC |
| MTDH-R | TCACGTTTCTCGTCTGGCTTTTTTC |

Table S2. Clinical characteristics of breast cancer patients

| **Patient**  **no.** | **Age**  **(year)** | **Tumor**  **size(cm)** | **Grade** | **TNM** | **ER** | **PR** | **HER2** | **MIB-1** | **Metastasis**  **Node count** | **Histological subtype** |
| --- | --- | --- | --- | --- | --- | --- | --- | --- | --- | --- |
| 1# | 37 | 3.0 | 2 | II | + | + | +++ | 30%+ | 2/13 | Ductal |
| 2# | 38 | 3.0 | 3 | III | - | - | +++ | 90%+ | 23/26 | Ductal |
| 3# | 38 | 3.0 | 3 | III | +++ | ++ | ++ | 70%+ | 22/25 | Lobular and colloid |
| 4# | 43 | 3.0 | 2 | II | ++ | +++ | + | 10%+ | 1/19 | Ductal |
| 5# | 45 | 6.0 | 3 | II | +++ | ++ | + | 40%+ | 13/22 | Ductal |
| 6# | 46 | 1.0 | 3 | II | + | + | ++ | 60%+ | 3/21 | Ductal |
| 7# | 46 | 2.5 | 3 | II | - | - | ++ | 70%+ | 0/21 | Ductal and Medullary |
| 8# | 48 | 2.5 | 3 | II | ++ | + | +++ | 15%+ | 0/4 | Ductal |
| 9# | 49 | 5.0 | 2 | II | + | - | + | 5%+ | 0/21 | Ductal |
| 10# | 49 | 3.5 | 2 | II | +++ | ++ | + | 10%+ | 1/21 | Ductal |
| 11# | 50 | 1.5 | 2 | I | +++ | - | ++ | 20%+ | 0/13 | Ductal |
| 12# | 50 | 3.0 | 3 | II | - | - | +++ | 15%+ | 4/10 | Ductal |
| 13# | 53 | 2.0 | 2 | I | +++ | +++ | + | 10%+ | 0/14 | Ductal |
| 14# | 54 | 2.5 | 2 | III | - | - | +++ | 40%+ | 23/30 | Ductal |
| 15# | 54 | 6.0 | 3 | III | - | - | - | 50%+ | 13/39 | Ductal |
| 16# | 54 | 2.5 | 3 | II | - | - | +++ | 25%+ | 0/11 | Ductal |
| 17# | 56 | 3.5 | 2 | II | +++ | - | +++ | 60%+ | 2/9 | Ductal |
| 18# | 56 | 5.0 | 2 | III | + | + | ++ | 10%+ | 14/14 | Lobular and colloid |
| 19# | 56 | 5.0 | 3 | II | + | - | ++ | 30%+ | 0/17 | Medullary |
| 20# | 57 | 2.5 | 3 | II | - | - | - | 85%+ | 0/17 | Ductal |
| 21# | 58 | 3.5 | 3 | II | - | - | +++ | 30%+ | 0/14 | Ductal |
| 22# | 59 | 2.5 | 3 | II | - | - | ++ | 80%+ | 0/13 | Ductal |
| 23# | 61 | 2.0 | 3 | III | +++ | +++ | + | 20%+ | 8/14 | Ductal |
| 24# | 61 | 0.7 | 2 | I | +++ | ++ | ++ | 10%+ | 0/12 | Ductal |
| 25# | 61 | 4.0 | 3 | II | - | - | ++ | 70%+ | 0/13 | Ductal |
| 26# | 63 | 2.8 | 3 | II | +++ | +++ | ++ | 10%+ | 3/15 | Ductal |
| 27# | 70 | 4.0 | 2 | II | ++ | + | + | 10%+ | 0/17 | Ductal |
| 28# | 75 | 2.0 | 2 | I | - | - | ++ | 40%+ | 0/16 | Ductal |
| 29# | 79 | 6.0 | 2 | III | +++ | +++ | - | 10%+ | 5/13 | Ductal |
| 30# | 81 | 4.0 | 3 | II | - | - | + | 80%+ | 0/16 | Ductal |
| 31# | 53 | 3.5 | 3 | II | +++ | + | - | 20%+ | 0/19 | Ductal |
| 32# | 49 | 2.0 | 2 | II | +++ | +++ | + | 5%+ | 0/15 | Ductal |
| 33# | 57 | 3.0 | 3 | II | +++ | + | +++ | 40%+ | 0/16 | Ductal |

Note: ER, estrogen receptor; PR, progesterone receptor; For the values of ER, PR and HER2, +++ stands for strongly positive, ++ and + stand for mildly positive, - stands for negative.

Table S3. Clinical characteristics of patients with breast cancer for tissue microarray study

| **No. of patients** | **Age (years)** | **TNM stage** | | | **Grade** | **Histological type** |
| --- | --- | --- | --- | --- | --- | --- |
|  |  | **T** | **N** | **M** |  |  |
| TM-1 | 36 | 3 | 3 | 1 | Ⅱ | Ductal |
| TM-2 | 38 | 3 | 1 | 0 | — | Lobular |
| TM-3 | 39 | 2 | 1 | 0 | Ⅱ | Ductal |
| TM-4 | 38 | 3 | 1 | 0 | Ⅱ | Ductal |
| TM-5 | 37 | 2 | 1 | 0 | — | Lobular |
| TM-6 | 78 | 2 | 1 | 0 | Ⅱ | Ductal |
| TM-7 | 46 | 3 | 0 | 0 | Ⅱ | Ductal |
| TM-8 | 53 | 3 | 1 | 1 | — | — |
| TM-9 | 48 | 2 | 1 | 0 | Ⅱ | Ductal |
| TM-10 | 38 | 2 | 1 | 1 | — | Tubular |
| TM-11 | 47 | 2 | 1 | 0 | Ⅱ | Ductal |
| TM-12 | 45 | 2 | 2 | 0 | Ⅱ | Ductal |
| TM-13 | 21 | — | — | — | Ⅱ | Ductal |
| TM-14 | 30 | 2 | 1 | 0 | Ⅱ | Ductal |
| TM-15 | 51 | 3 | 0 | 0 | Ⅱ | Ductal |
| TM-16 | 34 | 4 | 2 | 0 | Ⅲ | Ductal |
| TM-17 | 40 | 4 | 2 | 0 | Ⅲ | Ductal |
| TM-18 | 52 | 4 | 2 | 0 | Ⅱ | Ductal |
| TM-19 | 53 | 2 | 1 | 0 | Ⅲ | Ductal |
| TM-20 | 81 | 4 | 1 | 0 | Ⅱ | Ductal |
| TM-21 | 50 | — | — | — | Ⅲ | Ductal |
| TM-22 | 55 | 3 | 0 | 0 | Ⅱ | Ductal |
| TM-23 | 37 | 3 | 1 | 0 | Ⅲ | Ductal |
| TM-24 | 46 | 4 | 1 | 0 | Ⅱ | Ductal |
| TM-25 | 32 | 4 | 0 | 0 | Ⅱ | Ductal |
| TM-26 | 52 | 2 | 2 | 0 | Ⅰ | Ductal |
| TM-27 | 38 | 3 | 0 | 0 | Ⅰ | Ductal |
| TM-28 | 43 | 2 | 1 | 0 | Ⅲ | Ductal |
| TM-29 | 51 | 4 | 2 | 1 | Ⅱ | Ductal |
| TM-30 | 32 | 2 | 2 | 0 | Ⅱ | Ductal |
| TM-31 | 30 | 4 | 0 | 0 | Ⅱ | Ductal |
| TM-32 | 35 | 2 | 2 | 0 | Ⅱ | Ductal |
| TM-33 | 49 | 3 | 2 | 0 | Ⅱ | Ductal |
| TM-34 | 35 | 2 | 1 | 0 | Ⅱ | Ductal |
| TM-35 | 56 | 3 | 2 | 0 | Ⅱ | Ductal |
| TM-36 | 40 | 2 | 2 | 1 | Ⅱ | Ductal |
| TM-37 | 58 | 4 | 0 | 0 | Ⅱ | Ductal |

Note: “—” means missing data.
