# Supplementary figures and images for: MiR-128 suppresses metastatic capacity by targeting metadherin in breast cancer cells
Source: Biol Res. 2020 Sep 29;53:43. doi: 10.1186/s40659-020-00311-5 (PMC7526227; doi:10.1186/s40659-020-00311-5)

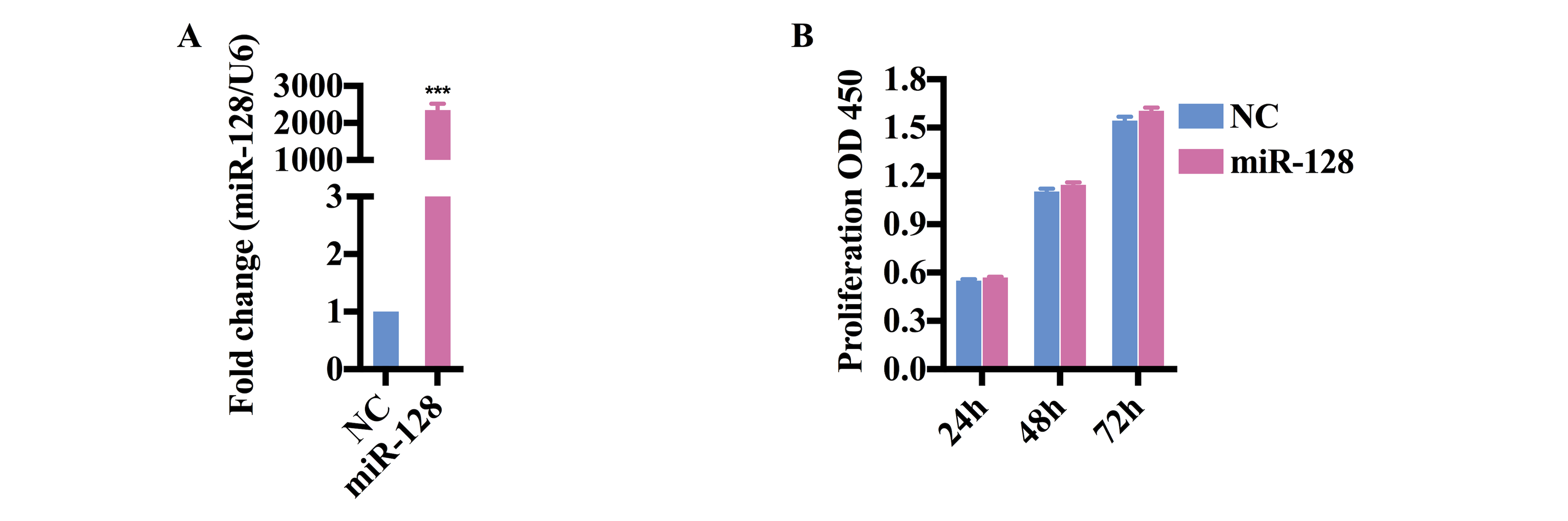

Supplement: Supplementary file 2 — Additional file 2: Fig. S1. (related to Fig. 2) Overexpression of miR-128 in breast cancer cell line MDA-MB-231 and cell viability assay after transfection. (A) Detection of miR-128 expression level in MDA-MB-231 cells transiently transfected with NC or miR-128 mimics. Bars represent the relative fold changes with U6 served as internal control. (B) Cell viability assay of MDA-MB-231 cells transfected with NC or miR-128 mimics for 24h, 48h and 72h. Bars represent the optical density at 450 nm. The symbol *** represents P < 0.001, using a two-tailed Student’s t-test. [file 40659_2020_311_MOESM2_ESM.tiff]

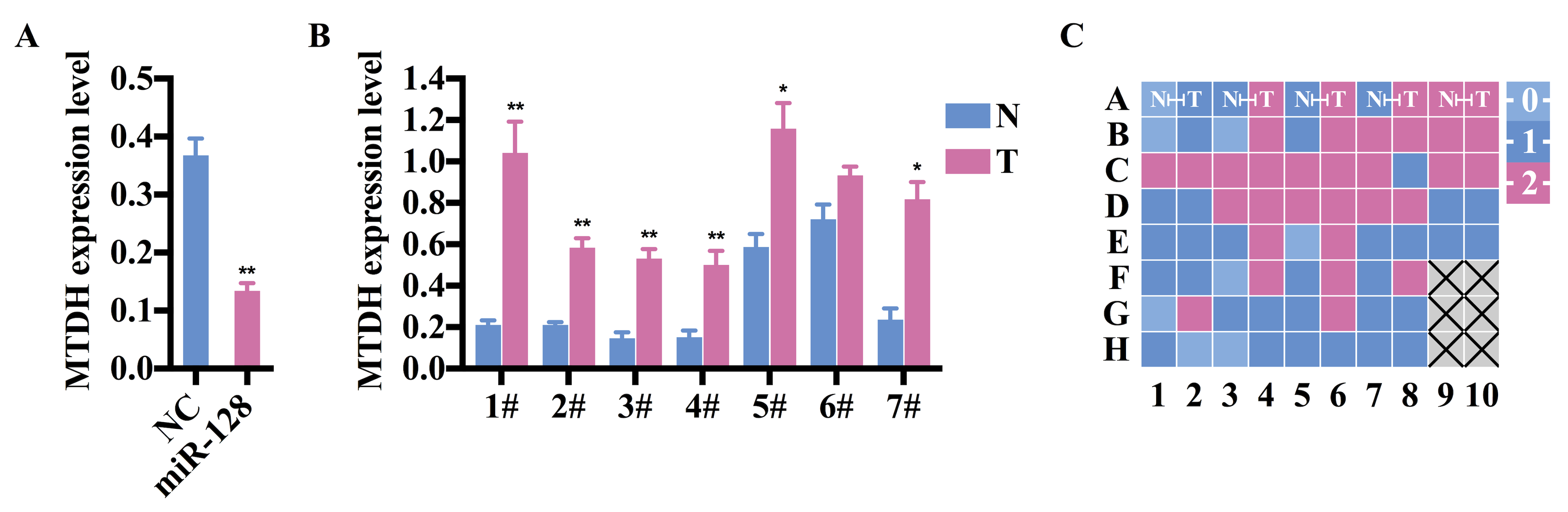

Supplement: Supplementary file 3 — Additional file 3: Fig. S2. (related to Fig. 3) Analysis of MTDH expression levels in breast cancer cell line, clinical specimens and tissue microarray. (A) The protein levels of MTDH in MDA-MB-231 cells transfected with NC or miR-128 mimics are normalized against β-actin and displayed with gray value. (B) The protein levels of MTDH in 7 paired clinical breast cancer specimens are normalized against β-actin and presented with gray value. (C) Tissue microarray for MTDH with 37 paired clinical breast cancer specimens embedded. N and T represent adjacent normal tissue and paired breast cancer specimen, respectively. The squares marked with soft blue (0) or blue (1) represent negative staining, while squares in red (2) represent positive staining. The symbol * and ** represent P < 0.05 and P < 0.01, respectively, using a two-tailed Student’s t-test. [file 40659_2020_311_MOESM3_ESM.tiff]

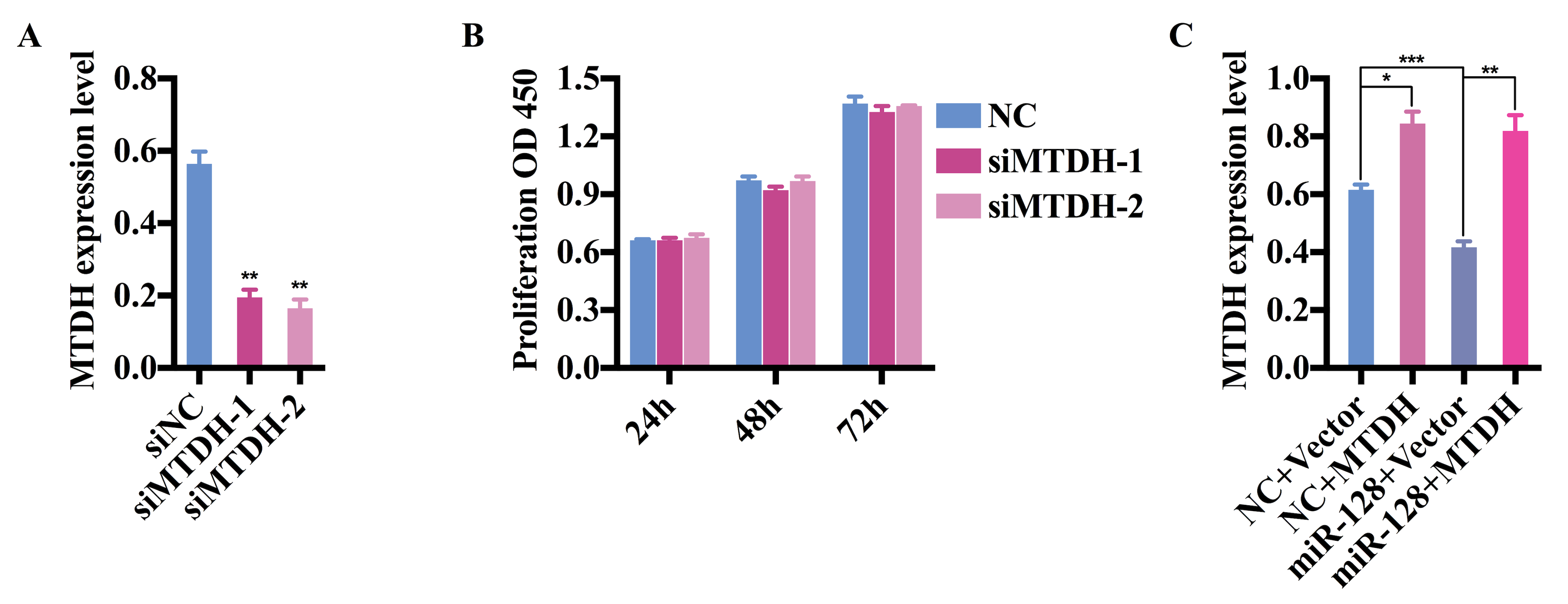

Supplement: Supplementary file 4 — Additional file 4: Fig. S3. (related to Fig. 4) Validation of MTDH knockdown and cell viability assay after MTDH silencing as well as analysis of MTDH expression levels after MTDH restoration. (A) The protein levels of MTDH in MDA-MB-231 cells transfected with siNC or siMTDH-1/2 are normalized against β-actin and and presented with gray value. (B) Cell viability assay of MDA-MB-231 cells transfected with siNC or siMTDH-1/2 for 24h, 48h and 72h. Bars represent the optical density at 450 nm. (C) The protein levels of MTDH in MDA-MB-231 cells cotransfected with NC or miR-128 mimics and pcDNA3.1-vector or pcDNA3.1-MTDH are normalized against β-actin and shown with gray value. The symbol *, ** and *** represent P < 0.05, P < 0.01 and P < 0.001, respectively, using a two-tailed Student’s t-test. [file 40659_2020_311_MOESM4_ESM.tiff]
